# Expression Profiles of GILZ and SGK-1 in Potentially Malignant and Malignant Human Oral Lesions

**DOI:** 10.3389/froh.2021.675288

**Published:** 2021-09-16

**Authors:** Mahmood S. Mozaffari, Rafik Abdelsayed

**Affiliations:** Department of Oral Biology and Diagnostic Sciences, The Dental College of Georgia, Augusta University, Augusta, Georgia

**Keywords:** human, GILZ, SGK-1, immunohistochemistry, leukoplakia, benign keratosis, epithelial dysplasia, squamous cell carcinoma

## Abstract

Glucocorticoid-induced leucine zipper and serum-glucocorticoid-regulated kinase-1 (SGK-1) are major glucocorticoid-inducible proteins. Recent studies indicate the local production of cortisol in oral mucosa, which can impact the tissue generation of glucocorticoid-induced leucine zipper (GILZ) and SGK-1. Furthermore, GILZ and SGK-1 play pathogenic roles in a variety of cancers, but their status in potentially malignant (e.g., epithelial dysplasia) or malignant oral lesions remains unknown. This study tested the hypothesis that expression profiles of GILZ and SGK-1, along with the phosphorylated (active) form of SGK-1 (pSGK-1), are different in epithelial dysplasia than squamous cell carcinoma. Accordingly, archived paraffin-embedded biopsy samples were subjected to immunohistochemistry to establish tissue localization and the profile of proteins of interest, while hematoxylin-eosin stained tissues were used for histopathological assessment. Based on histopathological examinations, tissue specimens were categorized as displaying mild-moderate or severe epithelial dysplasia and squamous cell carcinoma; benign keratosis specimens served as controls. All the tissue specimens showed staining for SGK-1 and pSGK-1; however, while SGK-1 staining was primarily cytoplasmic, pSGK-1 was mainly confined to the cell membrane. On the other hand, all the tissue specimens displayed primarily nuclear staining for GILZ. A semi-quantitative analysis of immunohistochemistry staining indicates increased GILZ expression in epithelial dysplasia but reversal in squamous cell carcinoma to a level seen for benign keratosis. On the other hand, the SGK-1 and pSGK-1 expressions decreased for squamous cell carcinoma specimens compared with benign keratosis or dysplastic specimens. Collectively, in this cross-sectional study, immunostaining patterns for proteins of interest do not seemingly differentiate epithelial dysplasia from squamous cell carcinoma. However, subcellular localization and expression profiles for GILZ, SGK-1, and pSGK-1 are suggestive of differential functional roles in dysplastic or malignant oral lesions compared with benign keratosis.

## Introduction

**Figure 1:**
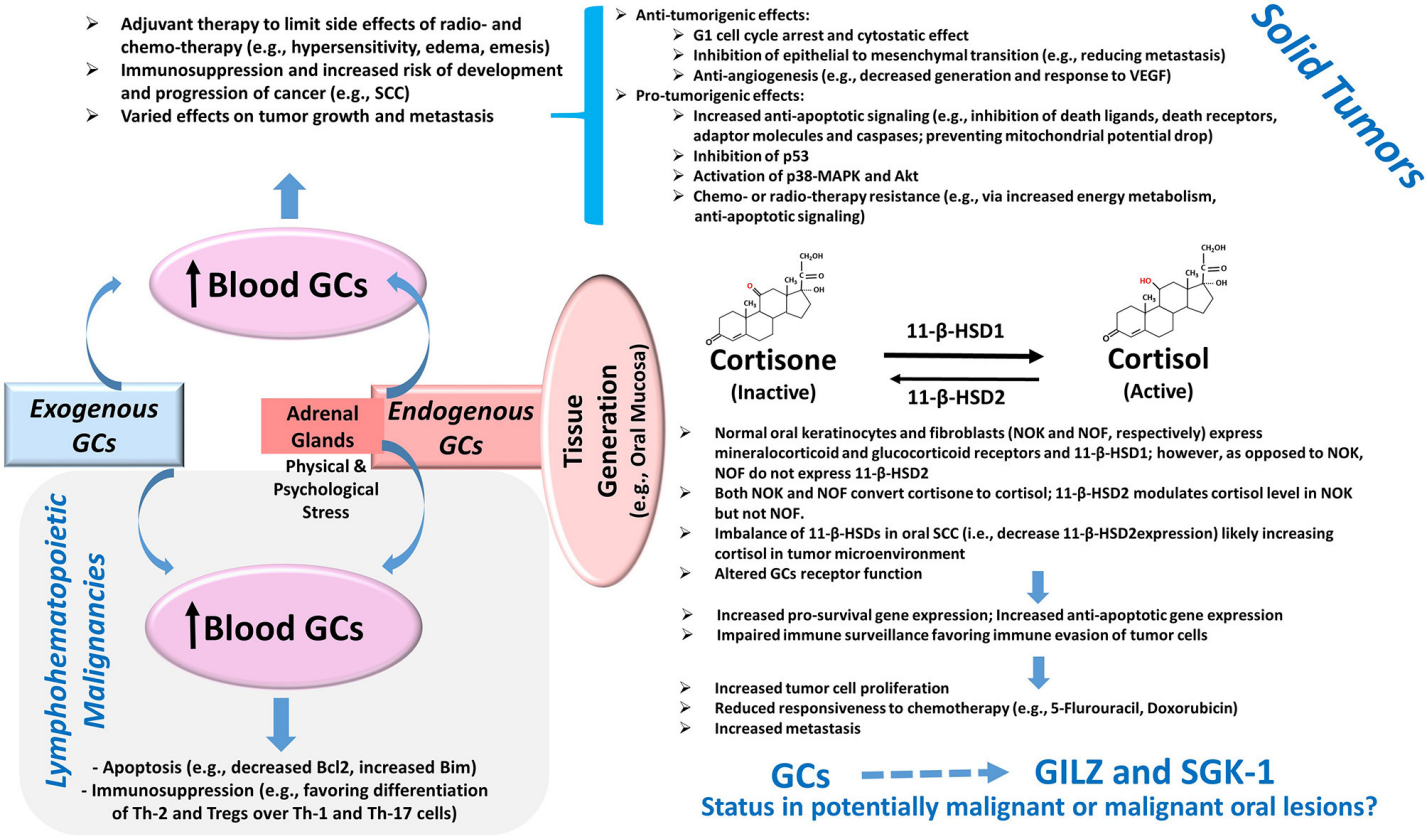
Glucocorticoids and Cancer. Exogenous administration of GCs and the production of GCs by adrenal glands cause an elevation in blood levels with consequent effects on tumors. The potent pro-apoptotic effects of GCs mediated in part *via* the modulation of the Bcl2 family of proteins as well as their immunomodulatory effects on Th cells and Tregs contribute to their beneficial effects in lymphohematopoietic malignancies. On the other hand, the use of GCs as adjuvant therapy in solid tumors can exert various effects on tumor growth and metastasis *via* a myriad of mechanisms, several of which are summarized in the diagram [2–6]. Importantly, more recent information suggests the presence of a non-adrenal GC system in several tissues including the oral mucosa. Accordingly, cortisol generation and its deactivation to cortisone occurs *via* tissue-specific 11-β-HSD1 and 2, respectively. The reduced expression of 11-β-HSD2 in oral SCC likely raises tissue cortisol level, resulting in glucocorticoid receptor-mediated effects. Consequently, it is proposed that the upregulation of pro-survival pathways coupled with impaired immune surveillance results in increased tumor size, reduced susceptibility to cytotoxic agents, and increased propensity for metastasis [10, 11]. Importantly, GCs are potent inducers of glucocorticoid-induced leucine zipper (GILZ) and serum-glucocorticoid-regulated kinase-1 (SGK-1) expressions, which are implicated in the pathogenesis of various tumors as described in the text; however, their status in potentially malignant or malignant oral lesions is not known and is the subject of this study. Abbreviations: 11-β-HSD, 11-β-hydroxysteroid dehydrogenase; GCs, glucocorticoids; MAPK, mitogen-activated protein kinase; SCC, squamous cell carcinoma; Th, T-helper cells; Tregs, regulatory T cells; VEGF, vascular endothelial growth factor.

Glucocorticoids are well-known for their immunosuppressive effects and are used in a variety of conditions with the hallmark feature of dysregulation of immune responses [1]. In oncology, the effects of glucocorticoids are context-specific and depend on a myriad of factors including tumor type, the expression level of glucocorticoids receptors, and tumor microenvironment [2]. Consequently, glucocorticoids are reported to either promote or suppress tumor growth *via* different mechanisms, some of which are itemized in Figure 1. For example, glucocorticoids serve as therapeutic options to promote apoptosis in lymphohematopoietic neoplasms but are also used as adjuvant therapy in non-hematological malignancies to limit the side effects of chemotherapy and radiotherapy [2–4]. Importantly, however, emerging information indicates that glucocorticoids can promote epithelial tumors *via* a number of mechanisms such as anti-apoptotic effects, impaired immune surveillance, increased energy metabolism of tumor cells, and conferred resistance to chemotherapy [4–6] (Figure 1). Indeed, patients with oral squamous cell carcinoma who are on immunosuppressant and glucocorticoid therapy show progression of cervical lymph nodes and extracapsular spread and are at increased risk for distant metastasis [7]. While adrenal glands are the major source of endogenous glucocorticoids, a growing body of evidence indicates that steroid metabolism occurs in tissues such as the colon, skin, and oral mucosa [8–10]. Tissue cortisol level is regulated by 11-β-hydroxysteroid dehydrogenases (11-β-HSDs) 1 and 2; 11-β-HSD1 metabolizes cortisone (inactive) to cortisol, which can be converted back to cortisone by 11-β-HSD2. Figure 1 summarizes some of the reported features of the glucocorticoid system in oral tissues [10, 11]. Indeed, the demonstration of a reduction in 11-β-HSD2 expression in oral squamous cell carcinoma (SCC) has focused attention on the role of local generation of glucocorticoids in oral tissue pathologies [10, 11].

Most effects of glucocorticoids are mediated *via* the regulation of glucocorticoid receptor gene transcription. Prominent among them are the glucocorticoid-induced leucine zipper (GILZ) and the serum-glucocorticoid-regulated kinase-1 (SGK-1), which were discovered as products of genes (*TSC22d3* and *SGK1*, respectively) whose expressions are markedly upregulated in response to glucocorticoids [1, 12, 13]. A myriad of physiological roles is attributed to GILZ and SGK-1, and their dysregulations are implicated in diverse pathological conditions such as cancer [1, 2, 14]. However, the status of GILZ and SGK-1 in potentially malignant or malignant lesions remains to be established.

Oral potentially malignant disorders include leukoplakia, which is a common lesion of the oral mucosa that appears as a white non-scrapable lesion that cannot be characterized clinically or pathologically as any other mucosal process [15–17]. Erythroplakia, likewise, is another oral potentially malignant disorder (OPMD) that presents as a solitary erythematous lesion of the oral mucosa [15, 18]. Importantly, clinical evaluation of leukoplakia or erythroplakia is usually followed by histopathological examination of tissue specimens to determine the presence and extent of epithelial dysplasia/carcinoma *in situ* or the development of invasive squamous cell carcinoma. Squamous cell carcinoma is the most common oral malignancy and typically develops from leukoplakia or erythroplakia [16–19]. It can present as white, erythematous, mixed white and erythematous, non-healing ulcerative- or mass-type lesion. The histopathological assessment of tissue specimen reveals a varying degree of differentiation with poorly differentiated lesions representing rapidly growing tumors with a high propensity for early metastasis.

The focus of this study is to establish the expression profiles of GILZ and SGK-1 and its phosphorylated, active, form (pSGK-1) in oral tissue specimens of human subjects, clinically found as leukoplakia, ulcerative or mass-type lesions, and diagnosed microscopically as epithelial dysplasia or squamous cell carcinoma. Aside from relevance to exogenous glucocorticoid therapy or the generation of glucocorticoids by adrenal glands, this investigation takes added importance given the demonstration that oral mucosa possesses the metabolic machinery to generate cortisol [10, 11] (Figure 1). Accordingly, GILZ, SGK-1, and pSGK-1 immunoprofiles and semi-quantitative assessment of staining in dysplastic and malignant lesions were contrasted with those assessed in benign keratosis specimens as controls.

## Methods

This study utilized archived paraffin-embedded biopsy specimens of patients who previously had presented for evaluation to the Department of Oral Biology and Diagnostic Sciences, Section of Oral Maxillofacial Pathology, of the Dental College of Georgia; personal identifiers of the patients were removed prior to the use of archived samples. These patients were initially evaluated for clinical presentations of their oral lesions followed by subsequent histopathological assessment of their biopsy specimens, which revealed mild-moderate epithelial dysplasia (*n* = 3), severe epithelial dysplasia (*n* = 3), and squamous cell carcinoma (*n* = 5); tissue specimens displaying benign keratosis (*n* = 5) were used as control. This study was considered exempt from review by the institutional review board. Table 1 summarizes the demographic information, anatomical site of lesion, and clinical impression/diagnosis of the subjects.

**Table 1:** Features of the experimental subjects whose biopsy samples were used for this study (SCC: squamous cell carcinoma).

**Features**				
**Subjects**	**Age (years)**	**Sex**	**Race**	**Anatomical site; clinical impression**
**Benign keratosis**				
Patient 1	50	Male	African-American	Lateral border of tongue; hyperkeratosis
Patient 2	64	Female	African-American	Posterior maxillary gingiva; hyperkeratosis
Patient 3	75	Female	Unknown	Posterior mandibular gingiva; hyperkeratosis
Patient 4	53	Male	Caucasian	Retromolar pad; tobacco keratosis
Patient 5	57	Female	Caucasian	Mandibular gingiva; hyperkeratosis vs. dysplasia
**Mild-Moderate Dysplasia**				
Patient 1	51	Male	Caucasian	Floor of mouth; snuff dipper's keratosis
Patient 2	70	Female	Caucasian	Lateral border of tongue; leukoplakia
Patient 3	44	Female	Caucasian	Buccal mucosa; leukoplakia
**Severe dysplasia**				
Patient 1	71	Female	Caucasian	Lateral border of tongue; leukoplakia; SCC
Patient 2	63	Male	Caucasian	Floor of mouth; dysplasia; SCC
Patient 3	66	Male	Caucasian	Ventral tongue; leukoplakia; SCC
**Squamous cell carcinoma**				
Patient 1	59	Female	Caucasian	Floor of mouth; SCC
Patient 2	64	Male	Caucasian	Floor of mouth; SCC
Patient 3	77	Female	Caucasian	Mandibular gingiva; SCC
Patient 4	51	Male	African American	Lateral border of tongue; SCC
Patient 5	91	Female	Caucasian	Palate; SCC

Paraffin-embedded tissue specimens were cut in 5-μm thickness, mounted on glass slides, and de-paraffinized in Leica Auto-Stainer XL (Leica, Wetzlar, Germany); antigen retrieval was performed using a citric acid-based antigen unmasking solution (Vector Laboratories, Burlingame, CA, United States). Tissue sections were then treated with 0.3% peroxidase for 30 min at room temperature, washed in water, and then incubated with a blocking solution (2.5% horse serum, 1% bovine serum albumin (BSA), and.5% Triton X-100) for at least 1 h at room temperature. Each primary antibody was diluted in the blocking solution (GILZ: 1:100; SGK-1: 1:100; pSGK-1: 1:50) and incubated with tissue sections overnight at room temperature; the GILZ mouse monoclonal antibody (LS-B4313) was obtained from LifeSpan Biosciences, Inc. (Seattle, WA, United States), the rabbit monoclonal antibody (ab32374) to SGK-1 was procured from Abcam (Cambridge, United Kingdom), and the pSGK-1 rabbit polyclonal antibody (Ser422) was purchased from Invitrogen (Waltham, MA, United States) (catalog number 44-1264G). The tissue sections were then washed two times in phosphate-buffered saline (PBS) and incubated for 1 h at room temperature with horseradish peroxidase-conjugated secondary antibody (Vector Laboratories, Burlingame, CA, United States) followed by 3,3′-diaminobenzidine (DAB) staining using the ImmPACT DAB Substrate Kit (Vector Laboratories, Burlingame, CA, United States). Slides were counter-stained with hematoxylin and mounted with a mounting medium. As a positive control, a human mammary tissue was used to establish staining for each protein of interest. For histopathological assessment, tissue specimens were subjected to H&E staining.

The semi-quantitative assessment of immunohistochemical staining utilized the Image J Fiji software and a previously described protocol [20]. The protocol involves deconvolution of immunohistochemistry images followed by the assessment of DAB staining, using mean gray intensity, and normalization to the nucleus. We also measured the fractional area of staining.

### Statistics

Semi-quantitative data are reported as means ± SEM for each condition. All the data were analyzed by analysis of variance followed by Newman–Keuls *post hoc* test to establish significance (*p* < 0.05) among the experimental conditions.

## Results

**Figure 2:**
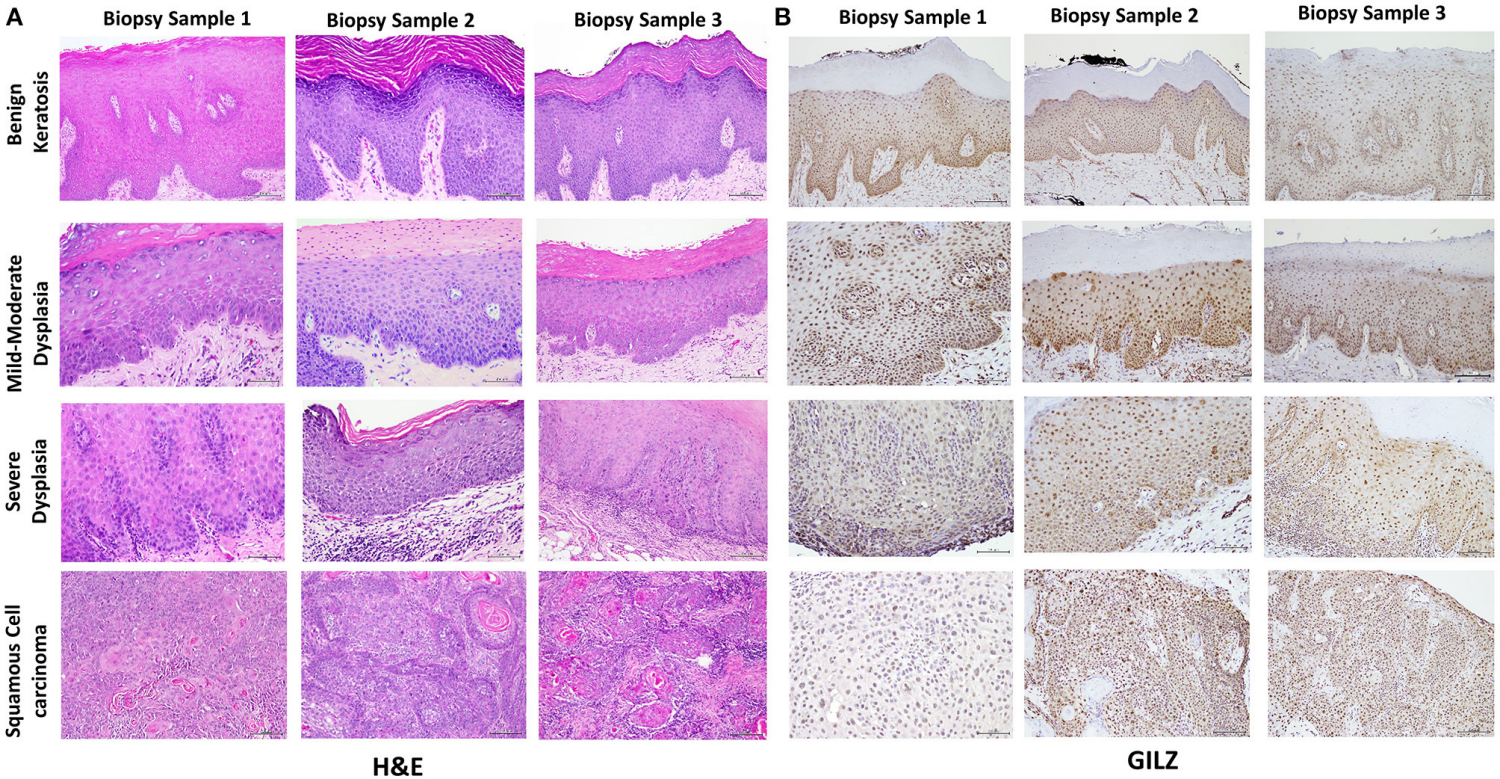
**(A)** shows hematoxylin-eosin (H&E) stained tissue specimens of the three subjects for each condition (×200). **(B)** shows glucocorticoid-induced leucine zipper (GILZ)-positive nuclear staining in benign keratosis as well as mild-moderate dysplasia and severe dysplasia throughout the full thickness of the epithelium, while GILZ-positive nuclear staining is observed throughout malignant epithelial cells (×200).

**Figure 3:**
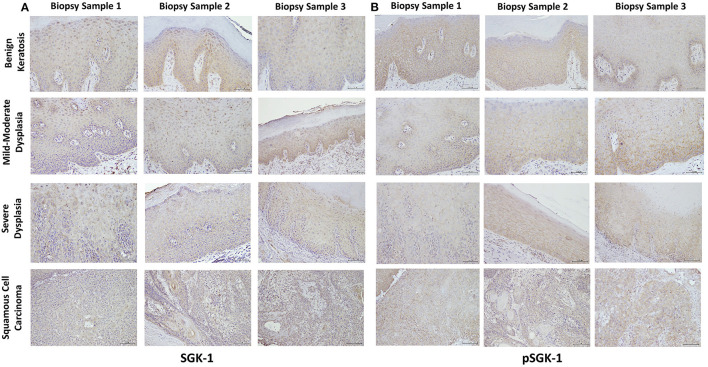
**(A,B)** show SGK-1 and phosphorylated SGK-1 (pSGK-1) immunostaining, respectively, for tissue specimens of the three subjects for each condition (×200). Benign keratosis, mild-moderate dysplasia, and severe dysplasia show prominent positive cytoplasmic staining and variable nuclear staining throughout the full thickness of the epithelium for SGK-1 **(A)**. It is noteworthy, however, that some cells did not show positive staining. The squamous cell carcinoma cases also manifested prominent cytoplasmic and some nuclear staining throughout the malignant epithelial cells for SGK-1 **(A)**. **(B)** shows that benign keratosis, mild-moderate dysplasia, and severe dysplasia display prominent positive cell membrane staining, without nuclear staining, throughout full thickness of the epithelium for pSGK-1. The squamous cell carcinoma cases exhibited positive cell membrane staining throughout malignant epithelial cells for pSGK-1 **(B)** ×200.

**Figure 4:**
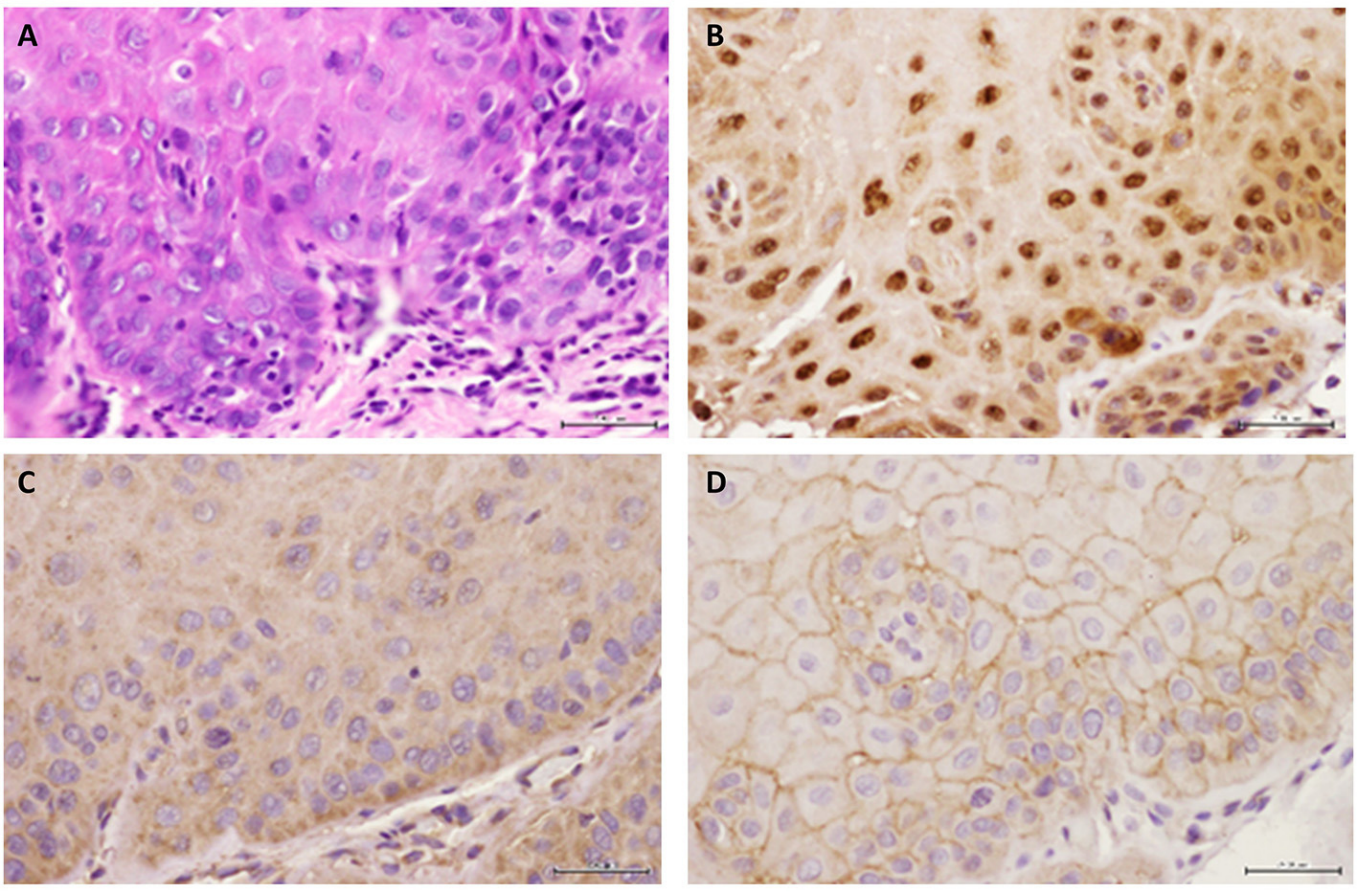
Representative images are shown for **(A)** hematoxylin-eosin, **(B)** GILZ, **(C)** SGK-1, and **(D)** pSGK-1 **(D)** for one case of severe epithelial dysplasia ×400.

Table 1 summarizes relevant features of the subjects whose biopsy specimens were used in this study. H&E staining for the tissue specimens is shown in Figure 2A, while the images for immunohistochemistry assessment of proteins of interest are displayed in Figures 2B,3; each figure shows 200 × images of the tissue specimens from three subjects for each condition. For greater ease in the identification of histopathological and immunohistochemical features, Figure 4 depicts ×400 images for one biopsy specimen in the category of severe dysplasia.

### Histopathology

Figure 2A shows histopathological features of specimens without evidence of dysplasia for the three subjects, with subject 1 displaying hyperparakeratosis and acanthosis and the other two cases manifesting hyperorthokeratosis. Specimens of the three subjects showed mild-to-moderate dysplasia with dysplastic epithelial changes being confined to the lower one-third-to-half of the epithelium; dysplasia was accompanied by hyperkeratosis in all the three cases (Figure 2A). Those manifesting severe epithelial dysplasia also included specimens from the three subjects for whom dysplastic epithelial changes involved at least three-quarter of the epithelial thickness; dysplasia was accompanied by hyperkeratosis in all the three cases (Figures 2A,4A). The cases of squamous cell carcinoma displayed invasive keratinizing squamous cell carcinoma showing individual cell keratinization and keratin pearl formation. Histologic grades ranged from well- to moderately differentiated carcinomas (Figure 2A).

### Glucocorticoid-Induced Leucine Zipper Immunohistochemistry

Benign keratosis specimens, without evidence of dysplasia, and those of mild-moderate and severe dysplasia showed positive nuclear staining throughout the full thickness of the epithelium for GILZ (Figures 2B,4B). Furthermore, the squamous cell carcinoma cases manifested positive nuclear staining throughout malignant epithelial cells (Figure 2B).

### Serum-Glucocorticoid-Regulated Kinase-1 and pSGK-1 Immunohistochemistry

Benign keratosis specimens, without evidence of dysplasia, and those of mild-moderate and severe dysplasia showed prominent positive cytoplasmic staining and variable nuclear staining throughout the full thickness of the epithelium for SGK-1 (Figures 3A,4C). It is noteworthy, however, that some cells did not show positive staining. Furthermore, the squamous cell carcinoma cases also manifested prominent cytoplasmic and some nuclear staining throughout malignant epithelial cells (Figure 3A).

Benign keratosis specimens, without evidence of dysplasia, and those of mild-moderate and severe dysplasia showed prominent positive cell membrane staining, without nuclear staining, throughout the full thickness of the epithelium for pSGK-1 (Figures 3B,4D). Furthermore, the squamous cell carcinoma cases exhibited positive cell membrane staining throughout malignant epithelial cells (Figure 3B).

### Staining Profile of Stromal Cells

Stromal components include fibroblasts, infiltrated white blood cells, cells of the vessel wall, and muscle cells. It is noteworthy that not all the tissue sections showed every stromal element or that staining for each protein was uniformly present for each cell type in each section of each category. Nonetheless, the examination of staining profiles revealed that GILZ and SGK-1 are not discriminatory among stromal elements, as indicated above, for each category, as staining was apparent for these cells albeit less for the muscle cells. Similarly, staining was present for pSGK-1 in white blood cells and cells of the vessel wall, but not muscle cells, of all the cases. However, the cases of benign keratosis and epithelial dysplasia, which were fewer than those of carcinoma, showed pSGK-1 staining for fibroblasts.

### Semi-quantitative Analysis

**Figure 5:**
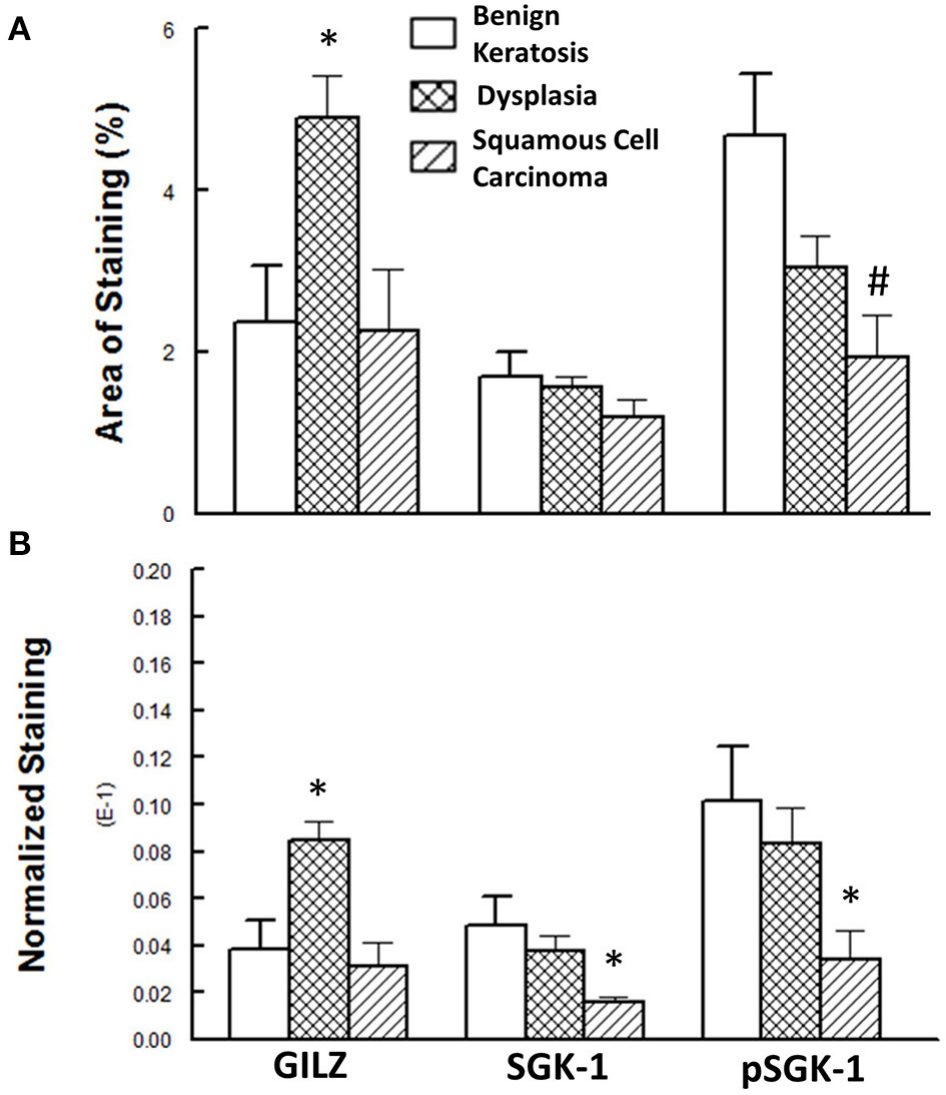
**(A)** Panel shows the fractional area of staining, while **(B)** panel shows staining normalized to nuclei for proteins of interest. Data are means ± SEM for each condition (*n* = 5–6 specimens for each condition as indicated under Methods). *p < 0.05 compared with the other two groups. #*p* < 0.05 compared with the benign keratosis group.

Figure 5 shows the results of the semi-quantitative assessment of staining for the experimental groups. For this assessment, data from mild-moderate dysplasia and severe dysplasia cases were pooled. As shown in panel A, the GILZ fractional area of staining was significantly greater for the dysplastic specimens compared with the other two categories, while the pSGK-1 staining area was significantly reduced for squamous cell carcinoma specimens compared with benign keratosis specimens. On the other hand, panel B shows DAB staining, normalized to the number of nuclei, as has been described in detail earlier [20], for the proteins of interest. Dysplastic lesions showed significantly greater GILZ staining compared with either the benign keratosis or squamous cell carcinoma specimens. Furthermore, the squamous cell carcinoma specimens displayed significantly reduced levels of SGK-1 and pSGK-1 compared with either benign keratosis or dysplasia specimens.

## Discussion

This study shows the subcellular localization and expression profiles for GILZ, SGK-1, and pSGK-1 of potentially malignant and malignant oral lesions. Accordingly, GILZ is primarily localized within the nucleus, while SGK-1 and pSGK-1 are primarily localized within the cytoplasm and the cell membrane, respectively. While the differential localization of proteins of interest is apparently not a major feature of potentially malignant and malignant oral lesions, or their stromal components, the semi-quantitative analysis of expression profiles suggests the differential regulation of GILZ compared with SGK-1 and pSGK-1 in this cross-sectional study. To the knowledge of the authors, this is the first report to establish the localization and expression profiles of two major glucocorticoid-regulated proteins in oral tissue specimens of human subjects, with heterogeneous demographic features, manifesting potentially malignant and malignant oral lesions. The observations take added importance given reports of the existence of a tissue-specific glucocorticoid system in the oral mucosa [10, 11] (Figure 1).

Inflammation plays an important role in initiating malignant transformation and promoting tumor growth and metastasis. Furthermore, inflammatory cells that infiltrate a tumor are important parts of the tumor microenvironment, affecting its growth and aggressiveness. Importantly, inflammation, immunomodulation, and cancer initiation and progression occur through common and overlapping pathways [2]. For example, nuclear factor kappa B (NF-κB) and activator protein 1 (AP-1) are correlated with the expression of genes that stimulate cancer cell proliferation and survival [21–23]. Furthermore, NF-κB interferes with p53, thereby conferring a potential tumor-promoting activity [24]. Thus, one can conjecture that since GILZ inhibits NF-kB and AP-1 transcriptional activity, it may curtail the development of tumor growth *via* the inhibition of pro-inflammatory cytokine production. However, concomitant GILZ-mediated immunosuppression may favor the development of tumors. Thus, similar to glucocorticoids, GILZ could exert cell- and context-specific tumor inhibition or promotion [2] (Figure 1). For example, GILZ inhibits Ras-dependent signaling pathways, a mechanism implicated in dexamethasone-induced antiproliferative effect on activated T lymphocytes and exerting antioncogenic activity *in vivo* and *in vitro* in Ras-transformed NIH-3T3 cells [25]. However, the GILZ expression in the cytoplasm of ovarian cancer cells, but not in the epithelium of normal ovaries and benign tumors, suggests its potential role in the proliferation of ovarian cancer cells [26]. Furthermore, utilizing an *in vitro* model with a cell line derived from ovarian cancer, it has been shown that GILZ increased Akt phosphorylation and activity, thereby enhancing proliferation [26]. In contrast, in estrogen-dependent MCF-7 breast adenocarcinoma cells, estrogen downregulates GILZ expression [27]. In dormant cells of murine melanoma, GILZ signaling antagonizes cell quiescence and induces cell cycle reactivation and tumor development, but GILZ repression induces cellular quiescence and contributes to melanoma inactivity [28]. High levels of GILZ have been associated with reduced responsiveness to cyclopamine therapy in lung cancer; cyclopamine is a Hedgehog pathway smoothened inhibitor, and the Hedgehog pathway is activated in lung cancer [29]. However, GILZ is reported to decrease the invasiveness of epithelial lung cancer cells *via* the inhibition of hypoxia-inducible factor-1α [30]. The influence of GILZ on cancer cell metabolism is also evident in studies indicating that GILZ overexpression induces a significant increase in mitochondrial oxidative phosphorylation associated with enhanced proliferation [31]. More recently, it has been shown that drugs targeting mitogen-activated protein kinase (MAPK) inhibit thyroid cancer cell proliferation in association with the upregulation of long-GILZ (L-GILZ; a variant of GILZ), which exhibits anti-oncogenic and antiproliferative activities [32]. Thus, the finding that L-GILZ binds to NF-κB and prevents its nuclear translocation in undifferentiated thyroid cancer cells has led to the suggestion that the L-GILZ-mediated trapping of NF-κB in the cytoplasm contributes to the inhibition of proliferation induced by drugs targeting the MAPK transduction cascade [32]. We observed nuclear staining for GILZ in human tissue specimens regardless of their clinical and histopathological presentations. However, the dysplastic specimens showed greater GILZ expression, an effect that was reversed for the squamous cell carcinoma specimens. The reason for the differential expression pattern for GILZ cannot be discerned from this study. However, given the complexity of GILZ actions as alluded to earlier and detailed recently by Ayroldi et al. [2], it is tempting to speculate a differential contribution of GILZ to dysplastic epithelial changes vs. squamous cell carcinoma growth and invasion, aspects that remain to be established.

The family of serine-threonine kinase consists of three members, with SGK-1 initially identified *via* the differential screening for glucocorticoid-inducible transcripts expressed in a rat mammary tumor cell line [13]. SGK-1 signals downstream of the phosphatidylinositol 3- kinase (PI3K) and shares similar structure, substrate specificity, and function with Akt [33]. The catalytic domain of SGK-1 is 54% identical to that of Akt, with which it shares a number of common downstream substrates. Similar to Akt, the phosphorylation of SGK-1 results in its activation and pro-survival activity [34, 35]. Importantly, SGK-1 has emerged as an important player in cancer biology, as exemplified by reports indicating increased expression and/or activity in several human tumors such as breast, tongue, head, and neck (e.g., squamous cell carcinoma), ovarian, prostate, multiple myeloma, and non-small cell lung cancer [36–43]. In addition, SGK-1 expression and activation contribute to tumor invasiveness, metastasis, and resistance to treatment [37, 44–46]. Given the pleiotropic role of SGK-1 in cancer biology, it has attracted much attention as a novel therapeutic target [47]. Indeed, a specific inhibitor of SGK-1 activity, SI113, induced cell death in a human colon carcinoma cell line and also potentiated paclitaxel sensitivity [48]. Furthermore, utilizing *in vitro* and *in vivo* models of hepatocarcinoma, it is reported that SI113 inhibits tumor growth [49]. Also, decreased SGK-1 mRNA expression accompanies a fluvastatin-induced reduction in resistance to chemotherapeutics and prevention of migration of breast and hepatocellular carcinoma cells [50]. Interestingly, we observed differential subcellular localization for SGK-1 and pSGK-1 in the human subject specimens; while SGK-1 is primarily localized within the cytoplasm, pSGK-1 displayed cell membrane localization. In this study, the reason for the localization pattern for the two forms of SGK-1 remains elusive. Nonetheless, it is noteworthy that a recent study utilizing HEK293 and opossum kidney tubule proximal cell lines concluded that the mTORC2-dependent phosphorylation of Ser422 (of SGK-1) occurs in the perinuclear membrane (while the phosphorylation of Akt occurs in the plasma membrane) [51]. Thus, it is likely that SGK-1 phosphorylation is context- and cell-specific. Another important finding relates to the observation of a reduction in the levels of both SGK-1 and pSGK-1 in the squamous cell carcinoma specimens compared with benign keratosis and dysplastic lesions, an effect more marked with respect to the former. Since pSGK-1 is the active and the pro-survival form of SGK-1, its reduction in human oral squamous cell carcinoma is suggestive of an adaptive mechanism to curtail tumor growth, an aspect of relevance in relation to the use of SGK-1 inhibitors as a therapeutic option in cancer [47].

Clearly, the use of archived human biopsy samples for research offers both opportunities and limitations. For example, the use of archived specimens facilitated the establishment of the link between lung cancer and smoking, the identification of *BRCA1* and *BRCA 2* genes, and progress in breast cancer research [52]. The limitations include the retrospective nature of the study, tissue samples coming from a single institute, small number of cases, inadequate information about the course and presentation of the lesions as well as other comorbid conditions, among others. With respect to this study, we acknowledge the relatively small sample size of the experimental groups as a limitation. Nonetheless, this aspect should be considered in the context of random case selection, heterogeneity of demographic information, site of lesion, and clinical impression of oral lesions.

In conclusion, despite the relatively small sample size, the results indicate the differential localization and expression of major glucocorticoid-regulated proteins, GILZ and SGK-1, in potentially malignant and malignant oral lesions. Given the complexities of GILZ and SGK-1 in tumor biology, coupled with the existence of a glucocorticoid system in the oral mucosa, elucidation of the mechanisms and contribution of each protein in these conditions would enhance the understanding of the pathogenesis of oral epithelial dysplasia and squamous cell carcinoma.

## Data Availability Statement

The original contributions presented in the study are included in the article/supplementary material, further inquiries can be directed to the corresponding author/s.

## Author Contributions

MM conceived the project, analyzed and interpreted the findings and wrote the manuscript. RA provided biopsy specimens, described histopathological and immunohistochemical observations and contributed to writing of the manuscript. All authors contributed to the article and approved the submitted version.

## Conflict of Interest

The authors declare that the research was conducted in the absence of any commercial or financial relationships that could be construed as a potential conflict of interest.

## Publisher's Note

All claims expressed in this article are solely those of the authors and do not necessarily represent those of their affiliated organizations, or those of the publisher, the editors and the reviewers. Any product that may be evaluated in this article, or claim that may be made by its manufacturer, is not guaranteed or endorsed by the publisher.
